# Sheathless High-Throughput Circulating Tumor Cell Separation Using Viscoelastic non-Newtonian Fluid

**DOI:** 10.3390/mi10070462

**Published:** 2019-07-10

**Authors:** Hyunjung Lim, Seung Min Back, Min Ho Hwang, Dae-Hee Lee, Hyuk Choi, Jeonghun Nam

**Affiliations:** 1 Department of Medical Sciences, Graduate School of Medicine, Korea University, Seoul 02841, Korea; 2 Graduate School of Medicine, Korea University College of Medicine, Seoul 02841, Korea; 3 Department of Oncology, Korea University Guro Hospital, Seoul 08308, Korea; 4 Department of Laboratory Medicine, College of Medicine, Korea University Guro Hospital, Korea University, Seoul 08308, Korea; 5 Department of Emergency Medicine, College of Medicine, Korea University Guro Hospital, Korea University, Seoul 08308, Korea

**Keywords:** circulating tumor cell, white blood cell, sheathless, high-throughput, viscoelastic fluid, separation

## Abstract

Circulating tumor cells (CTCs) have attracted increasing attention as important biomarkers for clinical and biological applications. Several microfluidic approaches have been demonstrated to separate CTCs using immunoaffinity or size difference from other blood cells. This study demonstrates a sheathless, high-throughput separation of CTCs from white blood cells (WBCs) using a viscoelastic fluid. To determine the fluid viscoelasticity and the flow rate for CTC separation, and to validate the device performance, flow characteristics of 6, 13, and 27 μm particles in viscoelastic fluids with various concentrations were estimated at different flow rates. Using 0.2% hyaluronic acid (HA) solution, MCF-7 (Michigan Cancer Foundation-7) cells mimicking CTCs in this study were successfully separated from WBCs at 500 μL/min with a separation efficiency of 94.8%. Small amounts of MCF-7 cells (~5.2%) were found at the center outlet due to the size overlap with WBCs.

## 1. Introduction

Circulating tumor cells (CTCs) are defined as rare tumor cells within the peripheral bloodstream. They are shed from the primary tumor, and can be a vital cause of hematogenous metastases [1,2]. Recently, CTCs have attracted increasing attention as they contain important information regarding cancer and its metastasis. CTCs can also be used as non-invasive biomarkers for early diagnosis, real-time monitoring of therapeutic processes, and genotypic and phenotypic changes in research applications [3,4]. However, due to their extreme rarity (1–10 CTCs per milliliter blood) and heterogeneity [5], it is difficult to distinguish CTCs amongst billions of red blood cells (RBCs) and millions of white blood cells (WBCs).

Recent advancements in microfluidics enable the utilization of microfluidic techniques for cell separation from a heterogeneous mixture sample [6,7]. Most current approaches for CTC separation employ affinity-based capture methods using an antibody that targets the tumor cell surface antigens, such as epithelial cell adhesion markers (EpCAM) [8]. However, due to the epithelial-to-mesenchymal transition (EMT), which may cause downregulation of epithelial surface markers, the capture efficiency can be limited [9]. To address the limitation of immuno-separation methods, size distinction of CTCs from other blood cells has been used as an alternative biophysical marker, negating the use of labeling. Microfluidic size-based separation techniques can be divided into two categories, active and passive, depending on the use of external forces. Active techniques employ external force fields, including dielectric [10,11] and acoustic fields [12,13,14,15], to separate CTCs, while passive methods utilize a special channel design, such as mechanical filtering techniques using microsieves [16,17], weir [18] and pillar arrays [19], deterministic lateral displacements (DLD) [20], dean flow fractionation (DFF) [21,22], and microfluidic vortices [23,24]. Active techniques for CTC separation have limited throughput due to the working time required for samples to be affected by external force fields (~20 μL/min [11] and ~80 μL/min [13]). Passive methods require no external force fields and can achieve relatively high throughput (~10 mL/min [20] and ~3 mL/min [17]), however, elaborate channel structures or complex fabrication processes for three-dimensional structures are required.

Recently, viscoelastic non-Newtonian microfluidics has gained heightened attention due to the intrinsic properties of viscoelastic fluids, which allow for easier manipulation of cells without the need for complex channel structures [25]. In addition, compared to previous passive methods, viscoelastic cell separation can be achieved over a wide working range of flow rates simply by modulating the viscoelastic fluid rheological properties. In a non-Newtonian fluid, non-uniform distribution of the first normal stress difference (*N*_1_) can drive suspended cells laterally in a simple straight microchannel, which has been applied to particle/cell focusing [26,27,28,29] and size-based particle/cell separation [30,31,32,33,34,35]. More recently, size-based separation techniques using viscoelastic fluid flows have been applied to separate CTCs from other blood cells [33,36,37]. However, the throughputs of these approaches (~7.5 μL/min [36] and ~50 μL/min [37]) were lower than previous reports on CTC separation (~80 μL/min [13], ~200 μL/min [12], and ~280 μL/min [38]), which can limit the separation of extremely rare CTC separation. Also, complicated channel geometry was required for high separation efficiency [33].

In this study, we show the sheathless, high-throughput separation of CTCs using a viscoelastic fluid in a low aspect ratio (AR = height/width) microchannel. Size-dependent equilibrium positions in a straight rectangular microchannel with a low AR have previously been examined using a viscoelastic fluid [37,38,39]. However, to the best of the authors’ knowledge, previous studies have not used a low AR microfluidic device for continuous separation of CTCs from lysed blood samples. We examined the equilibrium positions of particles with different sizes depending on the viscoelastic fluid rheological properties and flow rates. Finally, optimized flow characteristics with a specific flow rate and concentration of a viscoelastic fluid were adopted to separate CTCs. Based on the results provided in this work, our device provides a simple but powerful tool for high-throughput separation of extremely rare, heterogeneous CTCs.

## 2. Working Principle

**Figure 1:**
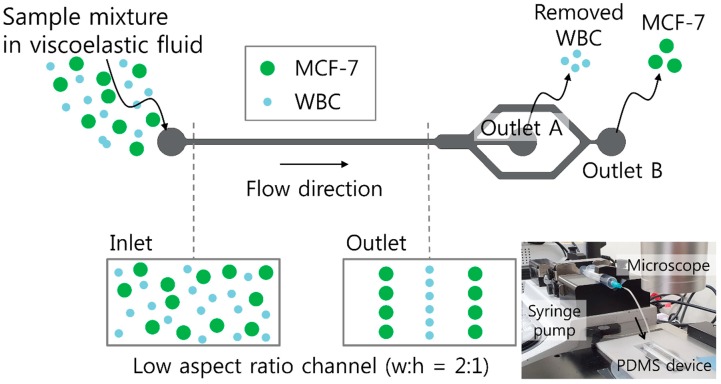
Schematic of sheathless, high-throughput separation of circulating tumor cells using a viscoelastic non-Newtonian fluid. Sample mixtures containing MCF-7 (Michigan Cancer Foundation-7) cells and white blood cells (WBCs) in a viscoelastic fluid were randomly introduced to the inlet of a low aspect ratio microchannel. Due to size-dependent viscoelastic separation, MCF-7 cells were separated at the rear outlet, while WBCs were removed at the center outlet. Inset (bottom right) shows an experimental setup consisting of a syringe pump, a microscope, and the polydimethylsiloxane (PDMS) channel device.

A schematic of the proposed device for sheathless high-throughput CTC separation using a viscoelastic non-Newtonian fluid is shown in Figure 1. As shown in the schematic, the device consists of a microfluidic channel with a low aspect ratio of 0.5. The initial sample mixture contains large tumor cells and relatively smaller WBCs suspended in a viscoelastic fluid with low viscosity but high elasticity [40]. This can enhance the throughput of the proposed device. At the inlet, the cells were injected as randomly distributed, as shown in the cross-sectional view in Figure 1.

During the flow of the viscoelastic fluid, the elastic force (*F_e_*) is induced by non-uniform differences in the first normal stress (*N*_1_), which depends on the cell size [41,42].

(1) $$F_{e}\sim a^{3}\frac{\partial N_{1}}{\partial x}\sim \lambda {\left(a/W\right)}^{3}Q^{3}$$

Here, *a*, *N*_1_, *x*, *W,* and *Q* are the cell diameter, first normal stress difference, lateral distance, width of the microchannel, and volumetric flow rate, respectively. The inertial lift force also affects the lateral migration of cells in a viscoelastic fluid, which can be divided into two counteracting components, namely, the shear gradient lift force (*F_i,s_*) and the wall repulsion force (*F_i,w_*).

(2) $$F_{i}=F_{i,s}+F_{i,w}\sim \rho {\left(a/W\right)}^{4}Q^{2}$$

Here, *ρ* is the solution density. The elastic force drives the suspended cells to low shear rate regions at the center and four corners of the microchannel. Conversely, the shear-gradient lift force drives the cells toward the channel walls, while the wall repulsion force drives cells to the center of the channel.

Both elastic and inertial forces affecting the suspended cells are dependent on the cell diameter, *a*, but elastic and inertial force scale differently with the cell diameter. The cells with different sizes therefore migrate toward distinct equilibrium positions in the microchannel, which enables size-based cell separation [38,39]. Briefly, the MCF-7 (Michigan Cancer Foundation-7) cells with a high blockage ratio (*β* = *a/H*; *H* indicates the channel height) are affected by the elastic force, driving the cells toward the channel walls. However, when considering small cells in comparison to the channel size (WBCs), inertial lift force affects the cells driving away from the channel walls and the center, while the elastic force drives the cells to the channel center. The diameters of WBCs and MCF-7 cells are known to be 9–15 μm and 14–27 μm, respectively [22,39]. To apply the working principle of our device for MCF-7 cell separation from WBCs, a straight microchannel with 100 μm width, 50 μm height, and 25 mm length was designed. Therefore, the blockage ratios (*β*) of WBCs and MCF-7 cells could be calculated as 0.18–0.3 and 0.28–0.54, respectively. As shown in the cross-sectional view at the outlet in Figure 1, WBCs are tightly focused at the center of the microchannel, while MCF-7 cells are located at two equilibrium positions between the center and the side walls of the microchannel. WBCs are removed from the initial mixture at the center outlet (Outlet A), while MCF-7 cells can be collected at the rear outlet (Outlet B). The inset figure at the bottom right shows the experimental setup of a syringe pump for sample injection, a microscope for optical observation, and the polydimethylsiloxane (PDMS) channel device with a single inlet and two outlets.

To characterize the fluid and the cell dynamics during the viscoelastic flow, non-dimensional numbers should be considered. The Reynolds number (*Re*) describes the ratio of the inertial force to the viscous force, $Re=\frac{\rho V_{m}D_{h}}{\eta_{c}}$, whereas the Weissenberg number (*Wi*) describes the ratio of the elastic force to the viscous force, $Wi=\lambda \dot{\gamma_{c}}$. Here, *ρ*, *V_m_*, *D_h_*, *η_c_*, *λ*, and $\dot{\gamma_{c}}$ indicate the solution density, mean flow velocity, hydraulic diameter of the particle, characteristic viscosity of the solution, fluid relaxation time, and characteristic shear rate, respectively. The relative effect of fluid elasticity to inertia is evaluated by the elasticity number, *El* = *Wi/Re*.

## 3. Materials and Methods

### 3.1. Device Design and Fabrication

A straight microfluidic channel of 100 μm width and 50 μm height was used, and thus the aspect ratio was defined as 1/2 (AR = height/width). The length of the main channel was 25 mm and the width of the expansion region at the outlet trifurcation was 800 μm for visualization of the flow streams of particles and cells. At the entrance region of the microchannel, micropillars of 50 μm width and 100 μm length were designed with 50 μm spacing to avoid the possible blockage of the microchannel by aggregated particles or CTC clusters. In clinical samples, CTC clusters can be found in approximately 5–20% of the total CTCs [43,44].

A polydimethylsiloxane (PDMS) microfluidic channel was fabricated using standard soft-lithography techniques with a replica mold, which was fabricated using an SU-8 negative photoresist (MicroChem, Newton, MA, USA) on a silicon wafer. A 10:1 mixture of the PDMS base and curing agent (Sylgard 184, Dow Corning, Midland, MI, USA) was cast over the replica mold, degassed in a vacuum chamber, and baked in an oven at 80 °C for 1 h. The cured PDMS channels were peeled off from the mold and bonded on a glass slide with oxygen plasma (CUTE, Femto Science, Gyeonggi, Korea). To minimize unwanted hydrophobic interactions between polystyrene particles and the channel surface, the PDMS channel was treated with Tween 20 [45].

### 3.2. Sample Preparation

Hyaluronic acid (HA) solutions at various concentrations (0.1, 0.2, and 0.3 (*w/v*) %) were prepared by adding hyaluronic acid (HA) sodium salt (357 kDa, Lifecore Biomedical, Chaska, MN, USA) powder to phosphate-buffered saline (PBS) to evaluate the effect of viscoelasticity on flow characteristics. To estimate the flow characteristics, fluorescent polystyrene particles with diameters of 6 μm, 13 μm, and 27 μm (ThermoFisher, Waltham, MA, USA) were used. The particle diameters of 13 and 27 μm were selected as analogues to white blood cells (WBCs) and MCF-7 tumor cells, respectively. The particles were suspended in HA solution at each concentration at a final concentration of approximately 1.2 × 10^6^ particles/mL.

A droplet of untreated human blood directly taken from a fingertip was used. The blood samples were lysed by 10×-diluted BD FACS (Fluorescence Activated Cell Sorter) lysis buffer (BD (Becton, Dickinson and Company) Biosciences, San Jose, CA, USA) and WBCs in lysed blood sample were stained using a fluorescent dye (SYBR Green).

Human breast adenocarcinoma cell line MCF-7 cells were used to mimic CTCs in this study. MCF-7 cells were maintained and propagated in DMEM (Dulbecco’s Modified Eagle’s Medium)/F12 supplemented with 10% fetal bovine serum (FBS, Gibco-BRL (Bethesda Research Laboratories)) and 1% penicillin/streptomycin (P/S, Gibco-BRL). The cells were cultured in 75-cm^2^ cell culture flasks (VWR Scientific Products, Bridgeport, NJ, USA) in a humidified atmosphere with 5% CO_2_ at 37 °C to 70–80% confluence. Final concentrations of cells were ~1.2 × 10^5^ MCF-7 cells/mL and ~1.8 × 10^5^ WBCs/mL, respectively. Final concentration of MCF-7 cells in the sample solution was set to be higher than that of a clinical sample for demonstration of the device performance [46].

### 3.3. Fluid Rheology Measurements

The rheological properties of HA solutions were measured by a dynamic light scattering system (Zetasizer ZSP, Malvern Instruments, Malvern, UK) using microrheology measurement at 20 °C, since HA solutions are polymer solutions with low viscosity and elasticity. Based on measurement of viscoelastic moduli, G’ and G”, the zero-shear viscosity and the relaxation time of 0.1 (*w/v*) % HA solution were measured as 0.89 mPa·s and 0.25 ms, respectively, which showed good agreement with the reported values in previous research [40]. Measured zero-shear viscosities and the relaxation times of the polymer solutions are summarized in Table 1.

### 3.4. Experimental Procedure

The flow rate of the sample solution was controlled by using a syringe pump (KDS210, KD Scientific, Holliston, MA, USA). During the experiment, particles and cells flowing in the microchannel were monitored by an inverted microscope (CKX41, Olympus, Tokyo, Japan with a high-speed camera (V611, Phantom, Wayne, NJ, USA) and a fluorescent camera (CS230B. Olympus, Tokyo, Japan).

## 4. Results and Discussion

**Figure 2:**
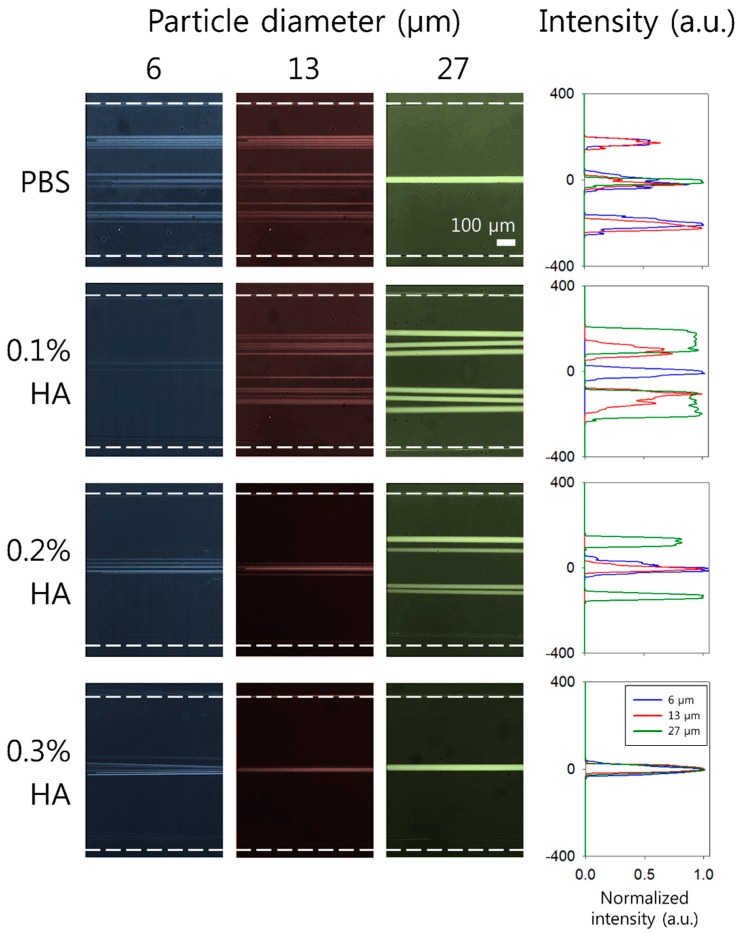
Effect of the polymer concentration on the particle size-dependent flow characteristics of fluorescent polystyrene particles with 6, 13, and 27 μm diameters using phosphate-buffered saline (PBS), 0.1%, 0.2%, and 0.3% HA solution. Stacked microscopic images (**left**) and normalized fluorescent intensities (**right**) in an expansion region at the fixed flow rate of 300 μL/min. White dotted lines indicate the channel sidewalls.

To examine the effect of viscoelasticity on flow characteristics of 6, 13, and 27 μm fluorescent particles (blockage ratios *β* = 0.12, 0.26, and 0.54), distributions of particles suspended in PBS, 0.1% HA, 0.2% HA, and 0.3% HA solution were observed. Figure 2 shows the stacked microscopic images and normalized fluorescence intensities of each particle in the expansion region at a flow rate of 300 μL/min. In PBS without elasticity, 6 and 13 μm particles (*β* = 0.12 and 0.26) were weakly focused into three fluorescent streams at the channel center and near the side walls, while 27 μm particles (*β* = 0.54) were tightly focused at the channel center at 300 μL/min (*Re* = 75.1). This agrees with previous reports regarding inertial flow characteristics in a low AR channel [47]. In 0.1% HA solution (*Re* = 74.9, *Wi* = 5.0, *El* = 0.06), 6 μm particles (*β* = 0.12) were focused along the centerline, while 13 and 27 μm particles (*β* = 0.26 and 0.54) were weakly focused into two fluorescent streams between the center and the side walls. For non-Newtonian fluid flow in a HA solution, the equilibrium position of particles was determined by the simultaneous effect of three main parameters: (1) flow inertia, (2) flow elasticity, and (3) the blockage ratio of each particle in the microchannel. For small particles compared to the channel size, inertial lift force drives particles away from the side walls and the center and elastic lift force drives particles to the center. Therefore, center-focusing of small particles can be achieved. When the blockage ratio of the particles is high (*β* > 0.25), the side wall-bound elastic lift force acts on the particles due to fluid elasticity, which shows a strong dependence on the particle size compared to the center-bound elastic force [38,48,49]. The particles with *β* > 0.25 are, therefore, off-center focused. These results showed good consistency with previous studies [38,39].

With an increased concentration of the HA solution to 0.2 (*Re* = 68.7, *Wi* = 5.6, *El* = 0.08) and 0.3% (*Re* = 57.5, *Wi* = 6.2, *El* = 0.10), *Wi* increases and *Re* decreases due to the increased relaxation time and viscosity, leading to increased *El*. As the elasticity is enhanced in 0.2% HA solution, the focusing positions of the particles (6 and 13 μm) were shifted to the center of the channel, while 27 μm particles were still focused into two streams at the equilibrium positions. In 0.3% HA solution, all particles (6, 13, and 27 μm) were tightly focused at the center of the channel. Flow patterns of 27 μm particles in PBS and 0.3% HA solution seemed to be identical, however, flow characteristics induced by inertial effect (PBS) and elastic effect (0.3% HA solution) were different, which can be seen in Figure S1.

**Figure 3:**
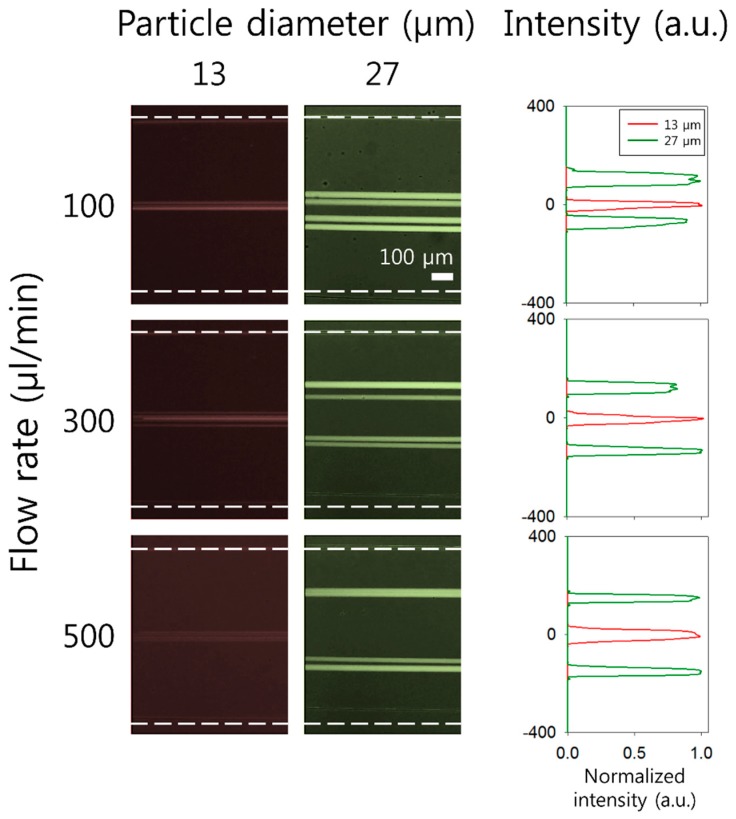
Effect of the flow rate on the particle size-dependent flow characteristics of fluorescent polystyrene particles with 13 and 27 μm diameters at the flow rates of 100, 300, and 500 μL/min. Stacked microscopic images (**left**) and normalized fluorescent intensities (**right**) of particles in an expansion region suspended in 0.2% HA solution. White dotted lines indicate the channel sidewalls.

Flow rate-dependent fluorescent particle distributions in the expansion region were examined at different flow rates of 100, 300, and 500 μL/min using 13 μm (*β* = 0.26) and 27 μm (*β* = 0.54) particles in 0.2% HA solution. Figure 3 shows normalized fluorescence intensities in the expansion region of 13 and 27 μm particles during the flow at 100, 300, and 500 μL/min, respectively. As the flow rate increases from 100 μL/min to 500 μL/min, the values of non-dimensional numbers (*Re* and *Wi*) increases, which results in a large elastic force. The focusing position of 13 μm particles remains identical over flow rates ranging from 100 μL/min (*Re* = 22.9, *Wi* = 1.87, *El* = 0.08) to 500 μL/min (*Re* = 114.5, *Wi* = 9.3, *El* = 0.08). Meanwhile, 27 μm particles that were focused near the center of the channel at 100 μL/min showed an outward migration into two separate streams as the flow rate increased to 300 and 500 μL/min. This shift of equilibrium positions may be explained by the strong dependence of side wall-bound elastic force on the flow rate and particle size. Based on this trend of 13 and 27 μm particles suspended in 0.2 % HA solution, our device can be applied to sheathless, label-free separation of cells.

**Figure 4:**
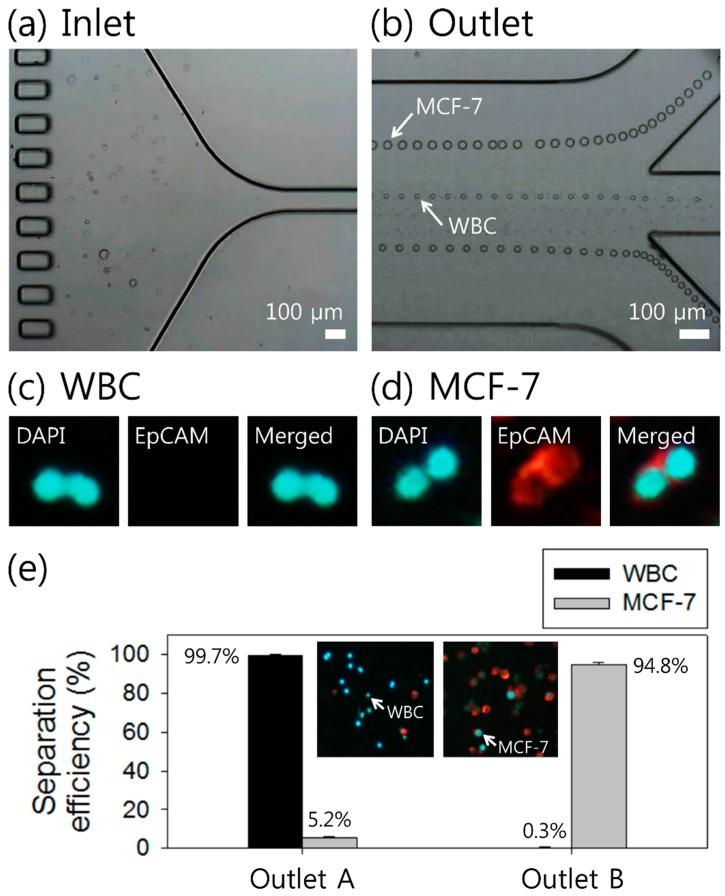
Cell separation of MCF-7 cells and WBCs at the flow rate of 500 μL/min using 0.2% HA solution. (**a**) At the inlet, both cells were randomly injected into the microchannel. (**b**) MCF-7 cells and WBCs were separated into two different streams along the center (WBCs) and off-center (MCF-7 cells). To identify (**c**) WBCs and (**d**) MCF-7 cells, fluorescent images and merged images were compared after staining with EpCAM and DAPI (4′,6-DiAmidino-2-PhenylIndole). (**e**) Separation efficiency at each outlet by using the collected sample after the separation process. Inset figures show the fluorescent images of cells collected at outlets A and B, respectively, after the separation process.

To evaluate the applicability of the device for clinical application of detecting CTCs in lysed blood samples, the separation of MCF-7 cells from WBCs was performed using optimized experimental conditions. The concentration of polymer solution (0.2% HA solution) and the flow rate (500 μL/min) were determined based on the results in Figure 3. Figure 4a,b shows the stacked microscopic images at the inlet and outlet of the microchannel during the separation process, which was recorded with a high-speed camera. A binary mixture containing both cells (MCF-7 cells and WBCs) was injected at the inlet without sheath fluids and the cells were randomly distributed in the microchannel, as shown in Figure 4a. At the outlet, MCF-7 cells and WBCs were separated into different streamlines, which flowed to different outlets (Figure 4b) (see supplementary video Movie S1). WBCs were tightly focused at the center of the channel due to their relatively small size (mean ± SD = 11.0 ± 5.0, SD is standard deviation, 0.12 ≦ *β* ≦ 0.32), while MCF-7 cells (mean ± SD = 23.1 ± 3.9, 0.38 ≦ *β* ≦ 0.54) migrated to two equilibrium positions between the channel center and the side walls, which shows good agreement with the flow characteristics of particles shown in Figure 2 and 3. Size distribution of both MCF-7 cells and WBCs can be found in Figure S2.

Figure 4c,d shows the fluorescent images of MCF-7 cells and WBCs after staining using DAPI (4′,6-DiAmidino-2-PhenylIndole) and EpCAM, respectively. EpCAM-phycoerythrin (PE (Phycoerythrin); red) was used as a surface marker for MCF-7 cells and a nuclear stain (DAPI; blue) was used to determine both MCF-7 cells and WBCs. Here, WBCs are negative to EpCAM. As a quantitative analysis of the device capability, separation efficiency was used, which can be defined as the ratio of the number of target cells at the target outlet to the total number of cells found at both outlets. As shown in Figure 4e, 99.7% of WBCs were collected at outlet A, while 94.8% of MCF-7 cells were collected at outlet B. This may be due to the wide size distribution of MCF-7 cells. The purity is defined as the ratio of the number of target cells to the total number of cells collected at the target outlet. The purity of MCF-7 cells collected at outlet B was approximately 98%. Inset figures show the fluorescent images of stained cells at outlet A and B collected after the separation. After the separation process, blue fluorescent cells were found in the samples collected from both outlets A and B, while only red fluorescent MCF-7 cells were collected from outlet B. After the separation process, cell viability was validated by the lactate dehydrogenase (LDH) assay, and no significant damage was observed in comparison with the cells before the separation process (Figure S3).

The breast cancer cells used for the current separation process are cultured MCF-7 cells (mean ± SD = 23.1 ± 3.9). The size distribution of MCF-7 cells and WBCs overlapped between 14 and 18 μm in diameter, in which 26% of WBCs and 7% of MCF-7 cells were included. Therefore, approximately 5.2% of MCF-7 cells were collected at outlet A along with WBCs, as shown in Figure 4e. In actual clinical samples from cancer patients, the size overlap of CTCs and WBCs can be even greater due to the cell heterogeneity [5]. To address the limitation of size overlap, microbeads that are coated with capture agent anti-EpCAM can be used to amplify the size difference between tumor cells and other blood cells for high-efficiency size-dependent separation in our proposed device [50].

The device throughput can be further enhanced to be greater than 500 μL/min by using a rigid plastic-based microfluidic device to address the present limitation of deformation of the PDMS microchannel at high flow rates. Microfluidic fittings and adapters can also be used for leak-tight connections. Moreover, because our device is a simple straight channel with a single inlet without any sheath flows, ultra-high throughput CTC separation can be achieved by stacking or multiplexing the devices [24,51,52]. In addition, compressed pneumatic pressure can be conveniently used for flow generation, negating the use of precise flow control system with external power source [27].

## 5. Conclusions

In summary, we described a slit microchannel with low AR for a sheathless, high-throughput cell separation device using a viscoelastic fluid. Polystyrene particles with different sizes of 6, 13, and 27 μm were used to evaluate the flow rate- and size-dependent flow characteristics in viscoelastic fluids with various concentrations (0.1, 0.2, and 0.3%). In 0.2% HA solution, particles smaller than 13 μm (*β* ≦ 0.26) were tightly focused at the center of the microchannel, while 27 μm particles (*β* = 0.54) were patterned into two fluorescent streams. Therefore, 13 and 27 μm particles in 0.2% HA solution can be separated at 500 μL/min, which was the optimized condition for CTC separation from WBCs. MCF-7 cells were finally separated with a 94.8% separation efficiency and 98% purity. Our slit microchannel device, therefore, enables the high-throughput separation process of extremely rare disease-related cells from a heterogeneous biological mixture sample.

## Figures and Tables

**Table 1:** Rheological properties of the prepared polymer solutions at 20 °C.

Properties	Hyaluronic Acid (HA) Concentration (wt.%)		
	0.1	0.2	0.3
Density (g/cm^3^)	1.0	1.0	1.0
Zero-shear viscosity (mPa·s)	0.89	0.97	1.16
Relaxation time (ms)	0.25	0.28	0.31

## Supplementary Materials

The following are available online at https://www.mdpi.com/2072-666X/10/7/462/s1. Figure S1: Flow characteristics of 6, 13 and 27 μm particles in PBS and 0.3% HA solution, respectively, depending on the flow rates. Figure S2: Initial size distribution of mixed sample of MCF-7 cells and leukocytes. Figure S3: The lactate dehydrogenase (LDH) assay that assess LDH released by MCF-7 cells before and after the separation process. After the separation in the viscoelastic fluid, no significant damage was found compared to the cells before the separation process (n.s. = not significant based on Student T-test). Video S1: Separation of MCF-7 cells from WBCs at 500 μL/min at the outlet.

## References

[1] Pantel K., Alix-Panabières C. (2010). Circulating tumour cells in cancer patients: Challenges and perspectives. Trends Mol. Med..

[2] Cristofanilli M., Budd G.T., Ellis M.J., Stopeck A., Matera J., Miller M.C., Reuben J.M., Doyle G.V., Allard W.J., Terstappen L.W.M.M. (2004). Circulating Tumor Cells, Disease Progression, and Survival in Metastatic Breast Cancer. N. Engl. J. Med..

[3] Alix-Panabieres C., Pantel K. (2016). Clinical Applications of Circulating Tumor Cells and Circulating Tumor DNA as Liquid Biopsy. Cancer Discov..

[4] Hou J.M., Krebs M., Ward T., Morris K., Sloane R., Blackhall F., Dive C. (2010). Circulating Tumor Cells, Enumeration and Beyond. Cancers.

[5] Song Y.L., Tian T., Shi Y.Z., Liu W.L., Zou Y., Khajvand T., Wang S.L., Zhu Z., Yang C.Y. (2017). Enrichment and single-cell analysis of circulating tumor cells. Chem. Sci..

[6] Shields C.W., Reyes C.D., Lopez G.P. (2015). Microfluidic cell sorting: A review of the advances in the separation of cells from debulking to rare cell isolation. Lab Chip.

[7] Cho H., Kim J., Song H., Shon K.Y., Han K.H. (2018). Microfluidic technologies for circulating tumor cell isolation. Analyst.

[8] Thege F.I., Lannin T.B., Saha T.N., Tsai S., Kochman M.L., Hollingsworth M.A., Rhim A.D., Kirby B.J. (2014). Microfluidic immunocapture of circulating pancreatic cells using parallel EpCAM and MUC1 capture: Characterization, optimization and downstream analysis. Lab Chip.

[9] Yang J., Weinberg R.A. (2008). Epithelial-mesenchymal transition: At the crossroads of development and tumor metastasis. Dev. Cell.

[10] Gascoyne P.R., Noshari J., Anderson T.J., Becker F.F. (2009). Isolation of rare cells from cell mixtures by dielectrophoresis. Electrophoresis.

[11] Shim S., Stemke-Hale K., Tsimberidou A.M., Noshari J., Anderson T.E., Gascoyne P.R. (2013). Dielectrophoresis has broad applicability to marker-free isolation of tumor cells from blood by microfluidic systems. Biomicrofluidics.

[12] Augustsson P., Magnusson C., Nordin M., Lilja H., Laurell T. (2012). Microfluidic, Label-Free Enrichment of Prostate Cancer Cells in Blood Based on Acoustophoresis. Anal. Chem..

[13] Antfolk M., Kim S.H., Koizumi S., Fujii T., Laurell T. (2017). Label-free single-cell separation and imaging of cancer cells using an integrated microfluidic system. Sci. Rep..

[14] Ding X., Peng Z., Lin S.C.S., Geri M., Li S., Li P., Chen Y., Dao M., Suresh S., Huang T.J. (2014). Cell separation using tilted-angle standing surface acoustic waves. Proc. Natl. Acad. Sci. USA.

[15] Li P., Mao Z., Peng Z., Zhou L., Chen Y., Huang P.H., Truica C.I., Drabick J.J., El-Deiry W.S., Dao M. (2015). Acoustic separation of circulating tumor cells. Proc. Natl. Acad. Sci. USA.

[16] Hosokawa M., Hayata T., Fukuda Y., Arakaki A., Yoshino T., Tanaka T., Matsunaga T. (2010). Size-Selective Microcavity Array for Rapid and Efficient Detection of Circulating Tumor Cells. Anal. Chem..

[17] Zheng S., Lin H.K., Lu B., Williams A., Datar R., Cote R.J., Tai Y.C. (2011). 3D microfilter device for viable circulating tumor cell (CTC) enrichment from blood. Biomed. Microdevices.

[18] Xu L., Mao X., Imrali A., Syed F., Mutsvangwa K., Berney D., Cathcart P., Hines J., Shamash J., Lu Y.J. (2015). Optimization and Evaluation of a Novel Size Based Circulating Tumor Cell Isolation System. PLoS ONE.

[19] Tan S.J., Lakshmi R.L., Chen P., Lim W.T., Yobas L., Lim C.T. (2010). Versatile label free biochip for the detection of circulating tumor cells from peripheral blood in cancer patients. Biosens. Bioelectron..

[20] Loutherback K., D’Silva J., Liu L., Wu A., Austin R., Sturm J.C. (2012). Deterministic separation of cancer cells from blood at 10 mL/min. AIP Adv..

[21] Warkiani M.E., Khoo B.L., Wu L., Tay A.K.P., Bhagat A.A.S., Han J., Lim C.T. (2016). Ultra-fast, label-free isolation of circulating tumor cells from blood using spiral microfluidics. Nat. Protoc..

[22] Kim T.H., Yoon H.J., Stella P., Nagrath S. (2014). Cascaded spiral microfluidic device for deterministic and high purity continuous separation of circulating tumor cells. Biomicrofluidics.

[23] Sollier E., Go D.E., Che J., Gossett D.R., O’Byrne S., Weaver W.M., Kummer N., Rettig M., Goldman J., Nickols N. (2014). Size-selective collection of circulating tumor cells using Vortex technology. Lab Chip.

[24] Che J., Yu V., Dhar M., Renier C., Matsumoto M., Heirich K., Garon E.B., Goldman J., Rao J., Sledge G.W. (2016). Classification of large circulating tumor cells isolated with ultra-high throughput microfluidic Vortex technology. Oncotarget.

[25] Lu X., Liu C., Hu G., Xuan X. (2017). Particle manipulations in non-Newtonian microfluidics: A review. J. Colloid Interface Sci..

[26] Leshansky A.M., Bransky A., Korin N., Dinnar U. (2007). Tunable Nonlinear Viscoelastic “Focusing” in a Microfluidic Device. Phys. Rev. Lett..

[27] Nam J., Jang W.S., Lim C.S. (2019). Non-electrical powered continuous cell concentration for enumeration of residual white blood cells in WBC-depleted blood using a viscoelastic fluid. Talanta.

[28] Ahn S.W., Lee S.S., Lee S.J., Kim J.M. (2015). Microfluidic particle separator utilizing sheathless elasto-inertial focusing. Chem. Eng. Sci..

[29] Kim B., Kim J. (2016). Elasto-inertial particle focusing under the viscoelastic flow of DNA solution in a square channel. Biomicrofluidics.

[30] Nam J., Lim H., Kim D., Jung H., Shin S. (2012). Continuous separation of microparticles in a microfluidic channel via the elasto-inertial effect of non-Newtonian fluid. Lab Chip.

[31] Nam J., Namgung B., Lim C.T., Bae J.E., Leo H.L., Cho K.S., Kim S. (2015). Microfluidic device for sheathless particle focusing and separation using a viscoelastic fluid. J. Chromatogr. A.

[32] Nam J., Shin Y., Tan J.K.S., Lim Y.B., Lim C.T., Kim S. (2016). High-throughput malaria parasite separation using a viscoelastic fluid for ultrasensitive PCR detection. Lab Chip.

[33] Nam J., Tan J.K.S., Khoo B.L., Namgung B., Leo H.L., Lim C.T., Kim S. (2015). Hybrid capillary-inserted microfluidic device for sheathless particle focusing and separation in viscoelastic flow. Biomicrofluidics.

[34] Liu C., Guo J., Tian F., Yang N., Yan F., Ding Y., Wei J., Hu G., Nie G., Sun J. (2017). Field-Free Isolation of Exosomes from Extracellular Vesicles by Microfluidic Viscoelastic Flows. ACS Nano.

[35] Faridi M.A., Ramachandraiah H., Banerjee I., Ardabili S., Zelenin S., Russom A. (2017). Elasto-inertial microfluidics for bacteria separation from whole blood for sepsis diagnostics. J. Nanobiotechnol..

[36] Tian F., Cai L., Chang J., Li S., Liu C., Li T., Sun J. (2018). Label-free isolation of rare tumor cells from untreated whole blood by interfacial viscoelastic microfluidics. Lab Chip.

[37] Liu C., Xue C., Chen X., Shan L., Tian Y., Hu G. (2015). Size-Based Separation of Particles and Cells Utilizing Viscoelastic Effects in Straight Microchannels. Anal. Chem..

[38] Li D., Lu X., Xuan X. (2016). Viscoelastic Separation of Particles by Size in Straight Rectangular Microchannels: A Parametric Study for a Refined Understanding. Anal. Chem..

[39] Nam J., Jang W.S., Hong D.H., Lim C.S. (2019). Viscoelastic Separation and Concentration of Fungi from Blood for Highly Sensitive Molecular Diagnostics. Sci. Rep..

[40] Lim E.J., Ober T.J., Edd J.F., Desai S.P., Heal D., Bong K.W., Doyle P.S., McKinley G.H., Toner M. (2014). Inertio-elastic focusing of bioparticles in microchannels at high throughput. Nat. Commun..

[41] Seo K.W., Byeon H.J., Huh H.K., Lee S.J. (2014). Particle migration and single-line particle focusing in microscale pipe flow of viscoelastic fluids. RSC Adv..

[42] Tehrani M. (1996). An experimental study of particle migration in pipe flow of viscoelastic fluids. J. Rheol..

[43] Aceto N., Bardia A., Miyamoto D.T., Donaldson M.C., Wittner B.S., Spencer J.A., Yu M., Pely A., Engstrom A., Zhu H. (2014). Circulating tumor cell clusters are oligoclonal precursors of breast cancer metastasis. Cell.

[44] Rostami P., Kashaninejad N., Moshksayan K., Saidi M., Firoozabadi B., Nguyen N. (2019). Novel approaches in cancer management with circulating tumor cell clusters. J. Sci. Adv. Mater. Dev..

[45] Zhou Y., Basu S., Wohlfahrt K.J., Lee S.F., Klenerman D., Laue E.D., Seshia A.A. (2016). A microfluidic platform for trapping, releasing and super-resolution imaging of single cells. Sens. Actuators B Chem..

[46] Geislinger T.M., Franke T. (2013). Sorting of circulating tumor cells (MV3-melanoma) and red blood cells using non-inertial lift. Biomicrofluidics.

[47] Zhou J., Giridhar P.V., Kasper S., Papautsky I. (2014). Modulation of rotation-induced lift force for cell filtration in a low aspect ratio microchannel. Biomicrofluidics.

[48] Dhahir S., Walters K. (1989). On Non-Newtonian Flow Past a Cylinder in a Confined Flow. J. Rheol..

[49] Huang P.Y., Joseph D.D. (2000). Effects of shear thinning on migration of neutrally buoyant particles in pressure driven flow of Newtonian and viscoelastic fluids. J. Non-Newtonian Fluid Mech..

[50] Liu H., Ao Z., Cai B., Shu X., Chen K., Rao L., Luo C., Wang F.B., Liu W., Bondesson M. (2018). Size-amplified acoustofluidic separation of circulating tumor cells with removable microbeads. Nano Futures.

[51] Khoo B.L., Warkiani M.E., Tan D.S.W., Bhagat A.A.S., Irwin D., Lau D.P., Lim A.S.T., Lim K.H., Krisna S.S., Lim W.T. (2014). Clinical Validation of an Ultra High-Throughput Spiral Microfluidics for the Detection and Enrichment of Viable Circulating Tumor Cells. PLoS ONE.

[52] Warkiani M.E., Khoo B.L., Tan D.S.W., Bhagat A.A.S., Lim W.T., Yap Y.S., Lee S.C., Soo R.A., Han J., Lim C.T. (2014). An ultra-high-throughput spiral microfluidic biochip for the enrichment of circulating tumor cells. Analyst.

