# Supplementary material for: Sheathless High-Throughput Circulating Tumor Cell Separation Using Viscoelastic non-Newtonian Fluid
Source: Micromachines (Basel). 2019 Jul 10;10(7):462. doi: 10.3390/mi10070462 (PMC6680956; doi:10.3390/mi10070462)
Supplement: Supplementary file 1 [file micromachines-10-00462-s001.zip › micromachines-538781 - sup - for final check/micromachines-538781 - sup - for final check.docx]

Supplementary Materials: Sheathless High-Throughput Circulating Tumor Cell Separation Using Viscoelastic non-Newtonian Fluid

Hyunjung Lim, Seung Min Back, Min Ho Hwang, Dae-Hee Lee, Hyuk Choi and Jeonghun Nam

Effect of Viscoelasticity on Flow Characteristics of Particles

Figure S1 shows the flow characteristics of 6, 13 and 27 μm particles in PBS and 0.3% HA solution, respectively. In PBS without elasticity, 6 and 13 μm particles (*β* = 0.12 and 0.26) were widely distributed in the microchannel at the flow rate of 100 μL/min and weakly focused into three fluorescent streams at the channel center and near the side walls at higher flow rates of 200 and 300 μL/min. However, 27 μm particles (*β* = 0.54) were tightly focused at the channel center at 100, 200 and 300 μL/min, which showed good agreement with the previous reports on the inertial flow characteristics in a low AR channel [1]. In 0.3% HA solution, all particles (*β* = 0.12, 0.26 and 0.54) were tightly focused along the centerline of the microchannel throughout the flow rate range (100, 300 and 500 μL/min).


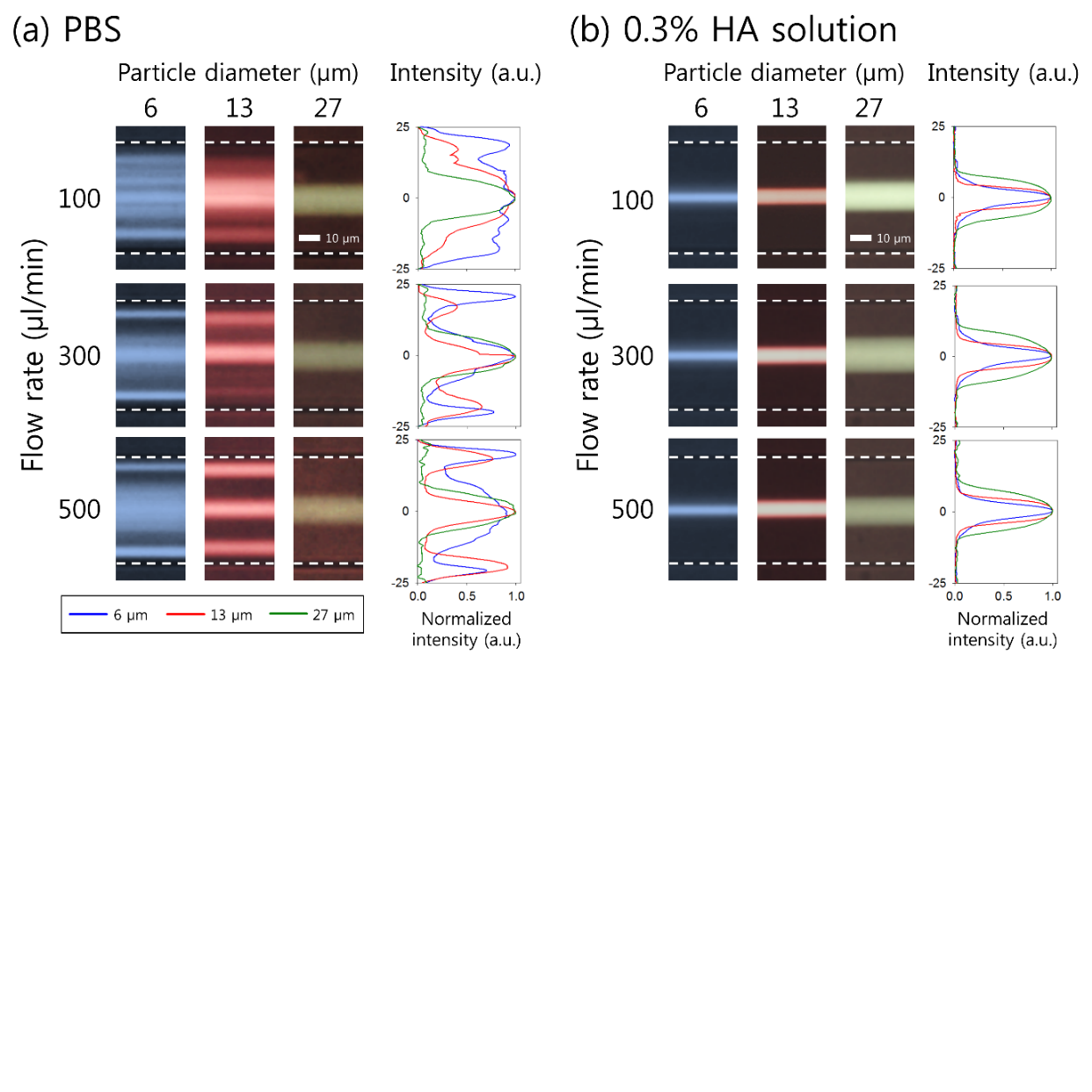


**Figure S1.** Flow characteristics of 6, 13 and 27 μm particles in PBS and 0.3% HA solution depending on the flow rates.

Size Distribution of Mixed Sample

Figure S2 shows the cell size distribution in the mixed sample before the separation process. WBCs attained after RBC lysis had a diameter ranging from 6 to 16 μm (mean ± SD = 11.0 ± 5.0, SD is standard deviation), whereas MCF-7 cells had a diameter range of 14–30 μm (mean ± SD = 23.1 ± 3.9). The size distribution of WBCs and MCF-7 cells overlapped between 14 and 18 μm, in which 26% of WBCs and 7% of MCF-7 cells were included. This can affect the separation efficiency of MCF-7 cells.


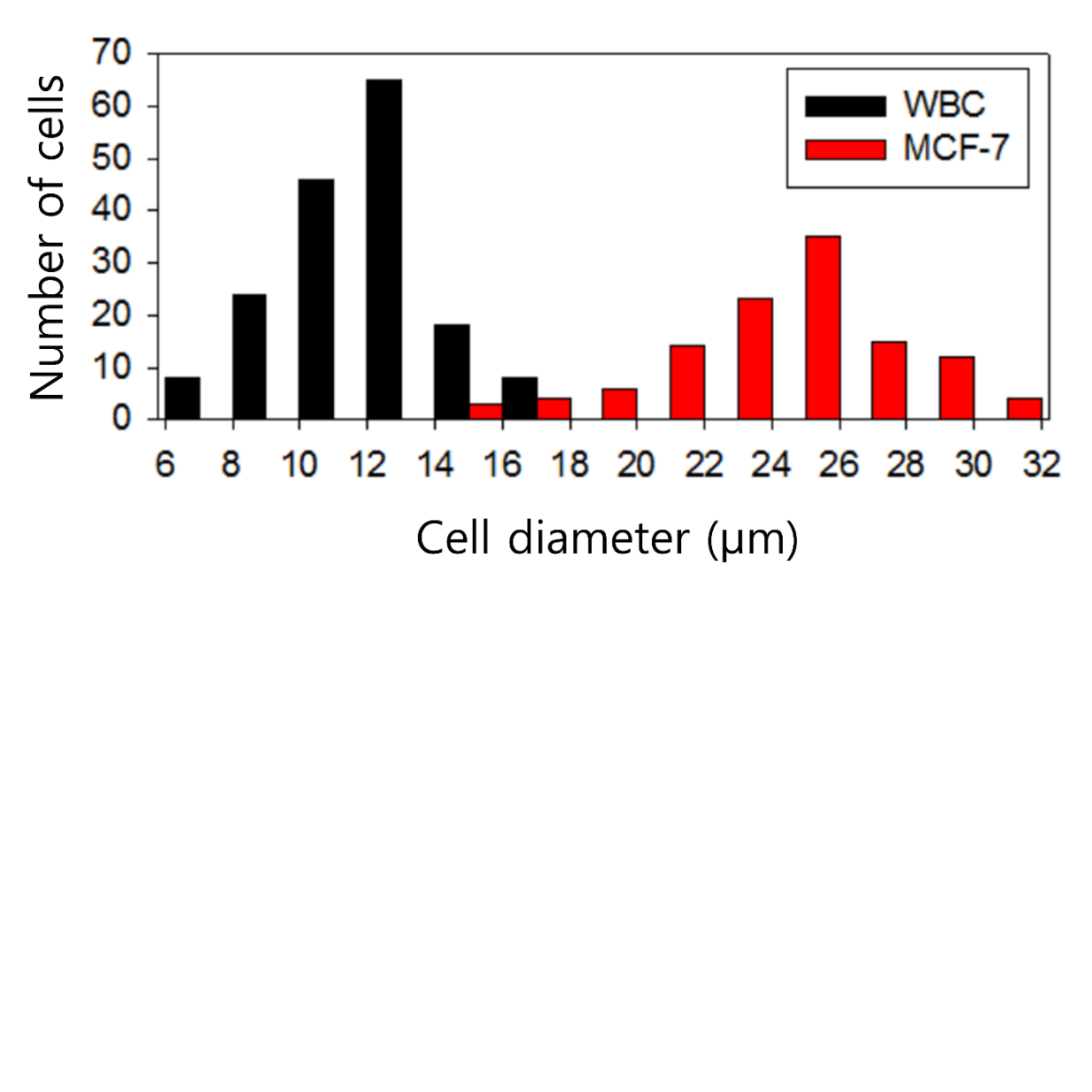


**Figure S2.** Initial size distribution of mixed sample of MCF-7 cells and leukocytes.

Assessment of the Cell Viability

Cell cytotoxicity was measured using Cytotoxicity Detection Kitplus LDH as described by the manufacturer. Lactate dehydrogenase (LDH) is a stable cytoplasmic enzyme present in cells. When the cells are damaged, it is released into the supernatant. In this study, the cells and supernatant were collected before and after the separation process for quantitating the release of LDH. Based on statistical analysis, there was no significant difference (Student's t-test, p = 0.086). Therefore, it can be concluded that the separation process using the viscoelastic fluid did not significantly compromise the cells’ viability.


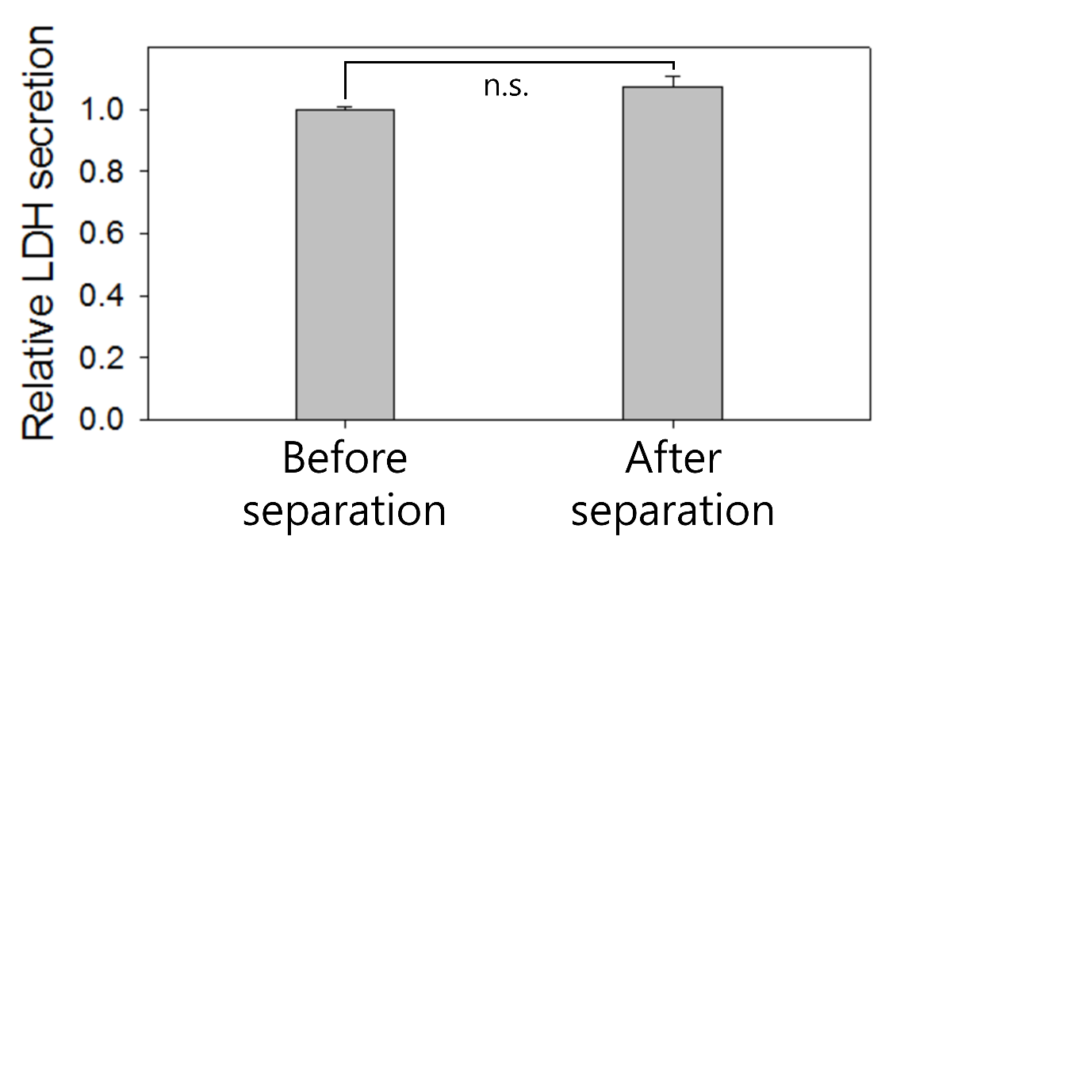


**Figure S3.** The lactate dehydrogenase (LDH) assay that assess LDH released by MCF-7 cells before and after the separation process. After the separation in the viscoelastic fluid, no significant damage was found compared to the cells before the separation process (n.s. = not significant based on Student T-test).

Reference

1. Zhou, J.; Giridhar, P.V.; Kasper, S.; Papautsky, I. Modulation of rotation-induced lift force for cell filtration in a low aspect ratio microchannel. *Biomicrofluidics*, **2014**, *8*, 044112
